# Tongue retraction using a McIvor blade improves airway condition during fiberoptic intubation: a randomized controlled trial

**DOI:** 10.1038/s41598-023-42503-5

**Published:** 2023-09-15

**Authors:** Jiyoun Lee, Sung-Hee Han, Jin-Hee Kim, Seongjoo Park, Ji Hyeon Lee, Hyeong Geun Kim, Jin-Woo Park

**Affiliations:** 1 Department of Anesthesiology and Pain Medicine, Seoul National University Bundang Hospital, 82 Gumi-ro, 173 Beon-gil, Bundang-gu, Seongnam, Gyeonggi-do 13620 Korea; 2 Department of Anesthesiology and Pain Medicine, College of Medicine, Seoul National University, Seoul, 03080 Korea; 3 Department of Anesthesiology and Pain Medicine, Korea University Guro Hospital, Korea University College of Medicine, Seoul, 08308 Korea

**Keywords:** Oral anatomy, Randomized controlled trials

## Abstract

Airway clearance is crucial for successful fiberoptic intubation. We hypothesized that tongue retraction using a McIvor blade could facilitate fiberoptic intubation. This randomized clinical trial aimed to compare intubation time and airway condition between the jaw thrust maneuver and tongue retraction with the McIvor blade during fiberoptic intubation. Ninety-four adult patients scheduled for elective surgery were randomly assigned to one of two groups. During fiberoptic intubation, airway clearance was secured by applying the jaw-thrust maneuver (J group) or by tongue retraction using the McIvor blade (M group). We assessed the total intubation time, number of attempts for tube advancement, and airway clearance at the soft palate and epiglottis levels. The total intubation time was significantly shorter in the M group than in the J group (*p* = 0.035). The number of attempts to advance the tube was significantly lower in the M group (*p* = 0.033). Airway clearance at the soft palate level was significantly better in the M group than in the J group (*p* = 0.027). Retracting the tongue with the McIvor blade demonstrated a better condition for fiberoptic intubation and shortened total intubation time compared with the jaw-thrust maneuver.

**Clinicalregistiration:** CRIS; http://cris.nih.go.kr (KCT0002392) registered 28/07/2017.

## Introduction

Managing difficult airways is one of the most essential and challenging tasks for anesthesiologists^1^. While different types of devices have been used to facilitate its management, many anesthesiologists consider fiberoptic intubation (FOI) the safest and gold standard technique for anticipated difficult tracheal intubation^2–5^.

Securing an open airway is important for successful FOI. In anesthetized patients with reduced muscle tone, a downward shift of the soft palate, tongue, and epiglottis close to the posterior pharyngeal wall results in narrowing of the oropharyngeal space. Partial or complete airway obstruction makes the bronchoscope difficult to be handle and located at the glottis^6,7^.

Clearing the airway during FOI involves several maneuvers, such as jaw-thrust and lingual traction, and airway devices, including intubating airways and direct laryngoscopes^6–9^. Jaw-thrust is a non-invasive and the simplest type of maneuvers, but it does not guarantee full airway clearance, especially at the soft palate level^6,10^. Lingual traction with Duval forceps also failed to produce clearance at the epiglottic level in many patients^6^. The use of an airway or laryngoscope may interfere with the bronchoscopic pathway or advancement of the tracheal tube^11^.

The McIvor blade is a tongue retractor with a thin blade and flat handle. It is widely used during pharyngeal surgeries, such as tonsillectomy, to secure the operation field by pressing the tongue body/base and retracting them forward. The blade has also been reported to facilitate laryngeal mask airway insertion by providing additional airway space^12^. We hypothesized that retracting the tongue using a McIvor blade would help secure an open airway and produce a superior condition for FOI in anesthetized patients. This randomized trial was designed to compare the two maneuvers; jaw-thrust and tongue retraction, with the McIvor blade based on intubation time, difficulty, and airway clearance during FOI at the induction of general anesthesia.

## Methods

### Study

The randomized clinical trial protocol was performed in accordance with Declaration of Helsinki and approved by the Institutional Review Board of Seoul National University Bundang Hospital (approval number: B-1704-393-004; approval date: 13 June 2017) and registered at the Clinical Research Information Service (CRIS; http://cris.nih.go.kr; registration number: KCT0002392; registration number: 28 July 2017) prior to patient enrolment. Written informed consent for participation was obtained from each patient. This manuscript adheres to the CONSORT guidelines. The study was conducted between August 2017 and January 2018 at Seoul National University Bundang Hospital.

### Patients

American Society of Anesthesiologists (ASA) physical status class I or II patients who were > 18 years of age and scheduled for elective surgery under general anesthesia requiring orotracheal intubation were enrolled in this study. Patients with ASA class ≥ III, dentures, craniofacial anomalies, history of cervical spine diseases, who were at risk for pulmonary aspiration, and who required awake intubation were excluded. Enrolled patients were randomly allocated to a group that underwent the jaw-thrust maneuver (J group) or the McIvor blade (M group) in a 1:1 ratio. Randomization was performed by an independent anesthesiologist using a computer-generated random number table (Random Allocation Software version 1.0; Isfahan University of Medical Sciences, Isfahan, Iran), 5 min before admission to the operating room. Group information concealed within an opaque envelope was opened just before FOI attempt.

### Anesthesia and intervention

The patients were positioned supine with their heads on a standard surgical pillow. Standard monitoring of vital signs included electrocardiography, non-invasive blood pressure measurement, and peripheral oxygen saturation measurement. After 3 min of denitrogenation with 100% oxygen, general anesthesia was induced with 1–1.5 mg kg^−1^ of propofol and target-controlled remifentanil infusion (effect site concentration: 3 ng ml^−1^). Rocuronium at 0.6 mg kg^−1^ was administered after the patient lost consciousness, and facial mask ventilation with 3% sevoflurane in oxygen was done. Following the disappearance of T1 (train-of-four stimuli), FOI was performed by an anesthesiologist with more than 5 years of FOI experience. A tracheal tube (Taperguard TM, Covidien, Mansfield, MA, USA), with an internal diameter of 7.5 mm for male patients and 7.0 mm for female patients, was threaded over a fiberoptic bronchoscope (series 11302 BDX; Karl Storz, Tuttlingen, Germany). The concave curvature of the tube was oriented anteriorly, and the bevel was oriented towards the patient’s left side.

**Figure 1:**
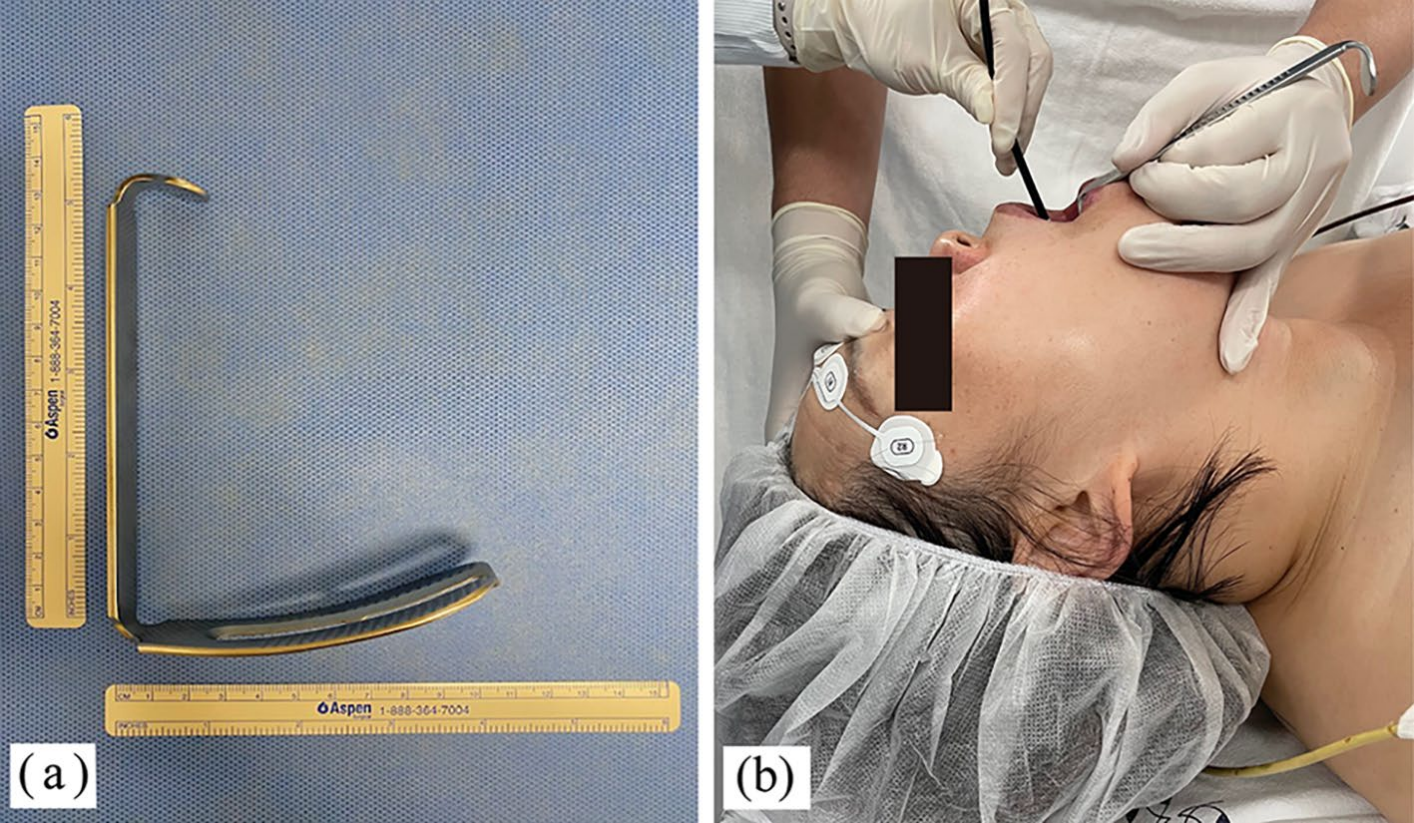
(**a**) The McIvor blade and (**b**) the placement of McIvor blade with the patient’s head extended. The participant provided consent for the publication of identifying image in an online open-access publication.

In the J group, an independent assistant in charge of airway maneuvers stood at the patient’s left side and applied a jaw-thrust maneuver prior to FOI. In the M group, a McIvor blade (size #4 from McIvor mouth gag set; Karl Storz GmbH, Tuttlingen, Germany) was placed with the patient’s head extended. The blade was fully inserted into the midline of the oropharyngeal space, placed at the base of the tongue, and gently lifted forward to clear the airway. This practice was performed by a maneuvering assistant who maintained the position of the patient’s head and continuously pulled the blade forward and upward (Fig. 1).

**Figure 2:**
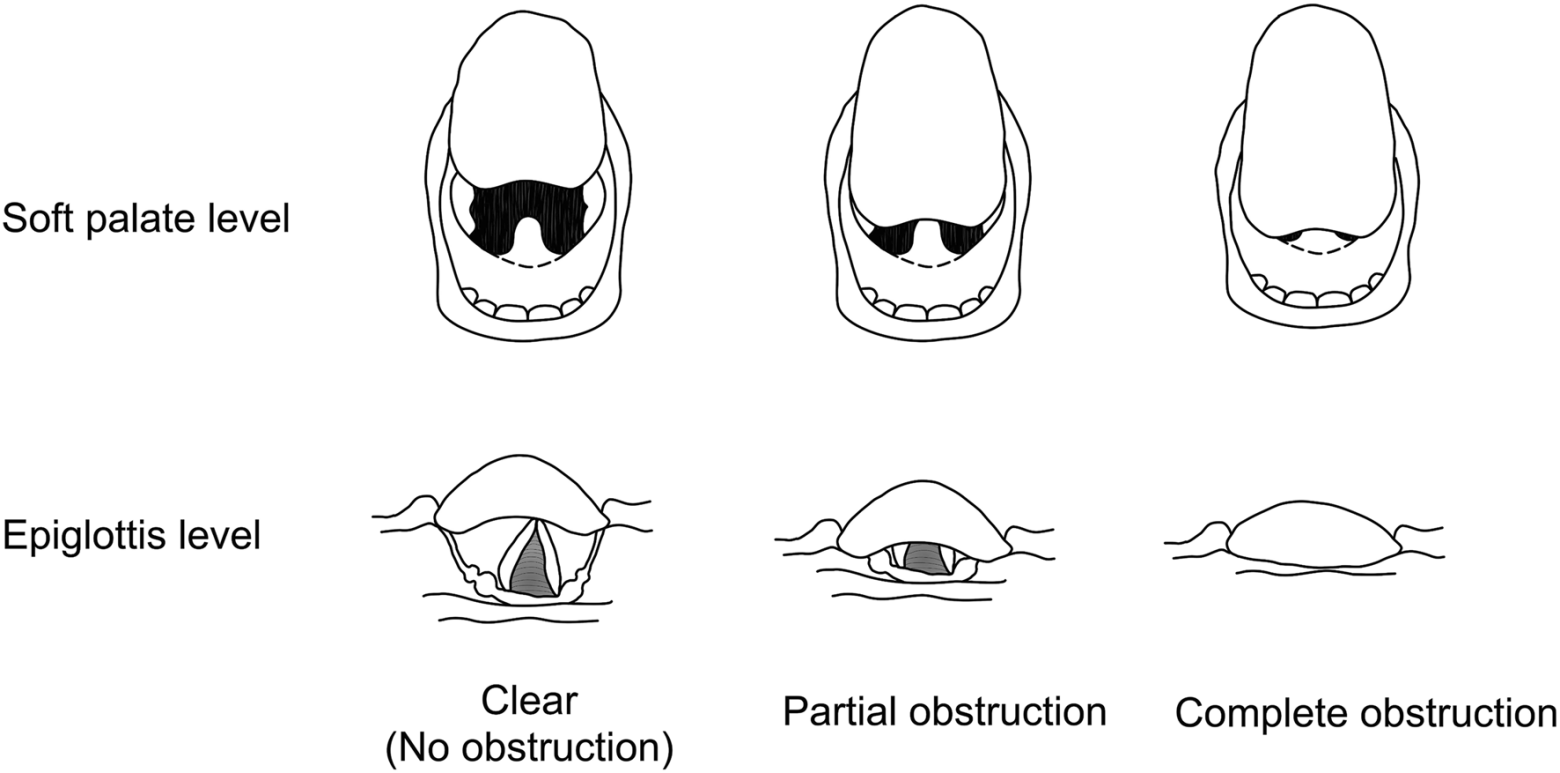
Bronchscopic assessment of airway clearance at the soft palate and epiglottis levels.

In performing FOI, the anesthesiologist inserted the bronchoscope into the mouth along the dorsum of the patient’s tongue and advanced it through the vocal cords. Airway clearance at the level of the soft palate was assessed by checking the position of the uvula or the soft palate from the dorsum of the tongue and was classified as clear (If the uvula did not touch the tongue), partially obstructed (If the uvula was in contact with the tongue), and complete obstruction (If the whole soft palate abutted the tongue). On the contrary, airway clearance at the epiglottis level was assessed by observing whether the epiglottis touched the posterior pharyngeal wall or not and was classified as clear (full view of the glottis), partially obstructed (If the sides of the epiglottis touched the posterior pharyngeal wall and there is a partial view of the glottis), and complete obstruction (If the glottis was not completely in sight) (Fig. 2).

If the approach to the vocal cord and carina was unsuccessful in 60 s or peripheral oxygen saturation decreased below 90%, two further trials were conducted after 2 min of mask ventilation with 3% sevoflurane in 100% oxygen. If successful insertion into the trachea was not performed after three trials, the FOI was recorded as a failure, and the anesthesiologist performed tracheal intubation using direct laryngoscopy.

After the fiberoptic bronchoscope entered the glottis and approached the carina level, the tracheal tube was advanced over the bronchoscope into the trachea, and the bronchoscope was removed to complete the FOI. If resistance was met during tube advancement, the tracheal tube was pulled out, rotated counterclockwise in steps of 90°, and advanced until it overcame resistance^13^. The airway clearing maneuver, jaw-thrust, or McIvor blade was maintained until tube advancement was completed. If attempts to advance the tracheal tube exceeded 60 s or peripheral oxygen saturation decreased below 90%, the FOI was recorded as a failure. The anesthesiologist removed the bronchoscope and performed tracheal intubation by direct laryngoscopy after 2 min mask ventilation with sevoflurane in oxygen.

After FOI was completed, the tracheal tube was connected to a ventilator circuit, and manual ventilation was attempted to confirm successful intubation with the presence of end-tidal carbon dioxide (EtCO_2_) waveform on the capnograph.

### Study outcome

The primary outcome was the total intubation time, defined as the duration from inserting the fiberoptic bronchoscope into the mouth to the appearance of EtCO_2_. The time required to approach the vocal cord and carina, the number of rotation attempts to complete the tube advancement, and airway clearance was recorded. Thyromental distance, inter-incisor gap, and Mallampati score were measured before the induction of general anesthesia by an independent anesthesiologist who was not involved in anesthetic induction and did not know the group information. The anesthesiologist also assessed postoperative sore throat using a numeric rating scale (NRS; 0, no pain; 100, worst pain) 1 h after surgery.

### Sample size

During a pilot study among 20 patients undergoing FOI intubation, the total intubation time was 26.5 (12.2) s when the jaw-thrust maneuver was applied (10 patients) and 20.4 (10.1) s when the McIvor blade was placed for tongue retraction (10 patients). Based on the pilot data, a sample size of 48 patients per group was required with a significance level of 95%, a power of 80%, and a 10% anticipated dropout rate.

### Statistical analysis

Continuous variables are presented as mean (standard deviation), and categorical variables are presented as numbers (percentages). All statistical analyses were performed using the SPSS version 21.0 software (SPSS Inc., Chicago, IL, USA). Student’s t-test was used to compare continuous variables, and the chi-squared test or Fisher’s exact test was used to analyze categorical variables. Statistical significance was set at a two-sided p value < 0.05.

## Results

**Figure 3:**
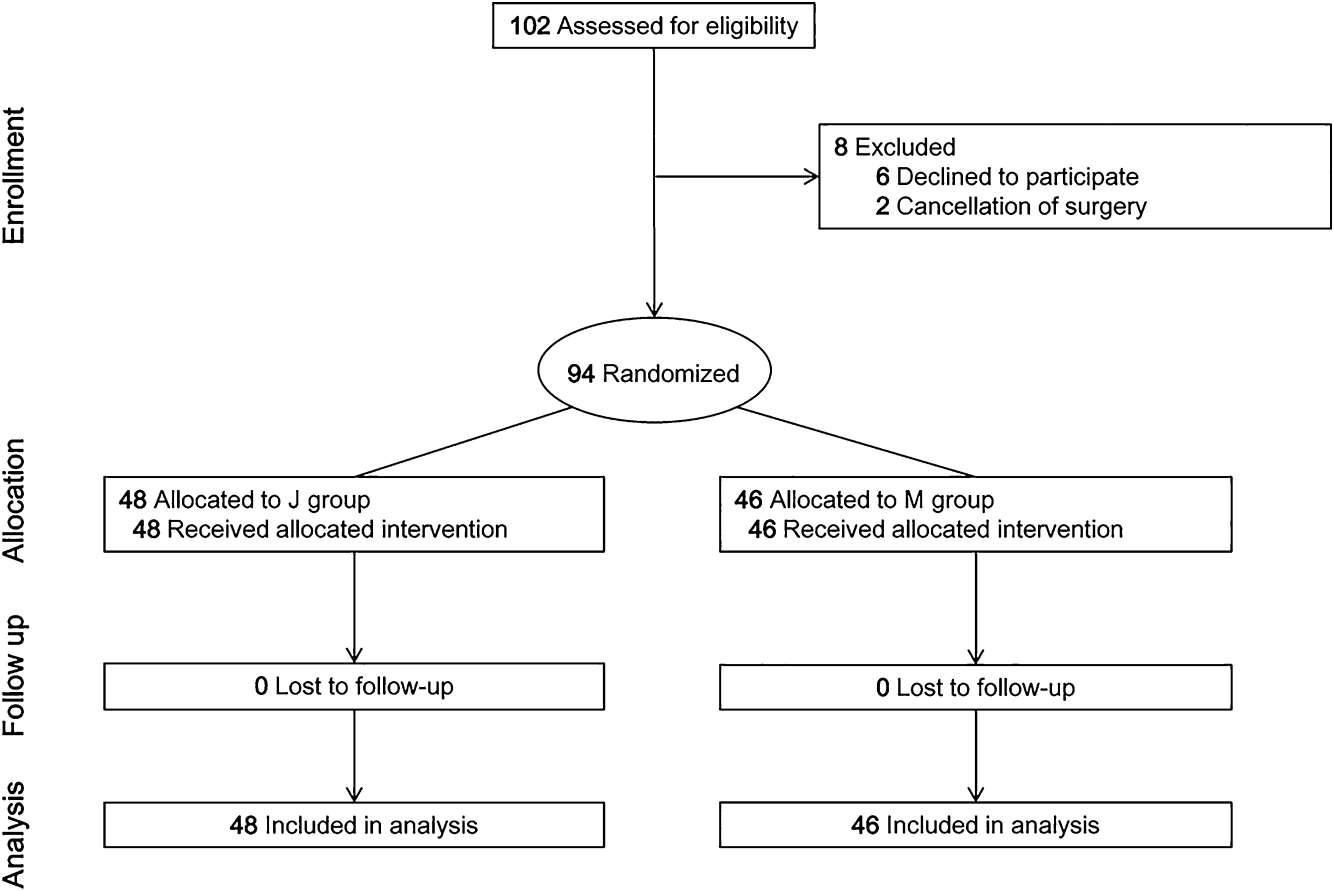
CONSORT flow diagram.

Initially, one hundred and two patients were included in this study. However, six patients refused to participate, and two were excluded due to surgery cancellations. Hence, ninety-four patients (J group: 48 and M group: 46) were enrolled (Fig. 3). Patient characteristics were not significantly different between the two groups (Table 1).

**Table 1:** Patients’ characteristics.

	J Group (*n* = 48)	M Group (*n* = 46)	*p* value
Age (year)	52.9 (13.0)	54.0 (13.1)	0.689
Height (cm)	162.2 (9.3)	158.0 (22.7)	0.245
Weight (kg)	64.0 (12.9)	62.6 (10.8)	0.582
Male	19 (39.6)	15 (32.6)	0.525
ASA (1/2)	22 (46)/26 (54)	22 (48)/24 (52)	1.000
TMD (cm)	8.7 (1.2)	9.1 (1.1)	0.121
Mouth opening (cm)	4.8 (0.7)	4.5 (0.7)	0.120
Mallampati (I/II/III/IV)	26(54.2)/15(31.3)/6(12.5)/1(2.1)	23(50.0)/14(30.4)/7(15.2)/2(4.3)	0.937

Values are presented as mean (standard deviation) or number (proportion) of patients.

*ASA *American Society of Anesthesiologists physical status, *TMD *thyromental distance.

Total intubation time was significantly shorter in the M group than in the J group (21.8 [16.3] vs. 27.6 [9.0]; mean difference (95% CI) 5.8 [0.4–11.2]; *p* = 0.035) (Table 2). Time taken to locate the bronchoscope in the vocal cord and carina was also shorter in the M group (mean difference (95% CI) 6.2 [3.0–9.4] for the vocal cord and 6.5 [3.1–9.8] for the carina; *p* < 0.001 for each comparison). A significant difference was also noted in the number of rotation trials for tube advancement between groups J and M (*p* = 0.033). For patients in the M group, there were no cases requiring tube rotation of 180° or more. In the J group, five patients required tube rotation ≥ 180° for tube advancement.

**Table 2:** Intubation data.

	J Group (n = 48)	M Group (n = 46)	Mean difference (95% CI)	*p* value
Time (s)				
EtCO_2_	27.6 (9.0)	21.8 (16.3)	5.8 (0.4 to 11.2)	0.035
Vocal cord	17.1 (8.7)	10.9 (6.7)	6.2 (3.0 to 9.4)	< 0.001
Carina	20.9 (9.0)	14.4 (7.0)	6.5 (3.1 to 9.8)	< 0.001
Number of 90° rotations for the tube advancement (0/1/2/3)	34 (73)/7 (13.0)/3 (6)/2 (4)	43 (91.3)/3 (8.7)/0 (0)/0 (0)		0.033
Failure of FOI	2 (4)	0 (0)		0.495
Degree of sore throat	26.9 (30.0)	21.1 (23.4)	5.8 (− 5.1 to 16.6)	0.292

Values are presented as mean (standard deviation) or number (proportion) of patients.

*EtCo2* end-tidal carbon dioxide, *FOI* fiberoptic intubation.

Two patients in the J group underwent tracheal intubation with direct laryngoscopy due to FOI failure. In one of the two patients, the insertion of the bronchoscope into the trachea could not be completed after three attempts; the insertion was achieved within the first 60 s in all other patients. In the other patient, tracheal tube advancement was unsuccessful even after a 60 s trial. Data from two patients were excluded from the analysis of intubation time and attempts of tube advancement. One hour after surgery, the degree of sore throat was not significantly different between the two groups.

Airway clearance at the soft palate level was significantly better in the M group than in the J group (*p* = 0.027) (Table 3). Three patients from the M group and thirteen from the J group showed partial or complete obstruction at the soft palate level. At the epiglottis level, airway patency was not significantly different among the two groups.

**Table 3:** Bronchoscopic classification of airway clearance.

	J Group (n = 48)	M Group (n = 46)	*p* value
Soft palate (clear/partial obstruction/complete obstruction)	35 (73)/9 (19)/4 (8)	43 (91)/2 (7)/1 (2)	0.027
Epiglottis (clear/partial obstruction/complete obstruction)	40 (83)/7 (15)/1 (2)	42 (91)/4 (9)/0 (0)	0.436

Values are presented as number (proportion) of patients.

## Discussion

In this randomized controlled study, we demonstrated that tongue retraction by applying the McIvor blade shortened the total intubation time, improved airway clearance, and facilitated tube advancement during FOI in comparison with the jaw-thrust maneuver. To the best of our knowledge, this study is the first trial to utilize a McIvor blade to enhance the airway condition for FOI.

The FOI plays an essential role in airway management. Awake intubation using a fiberoptic bronchoscope is the safest technique when a difficult airway is anticipated^5,7,11,14^. For patients requiring minimal cervical movement, FOI enables more meticulous airway management than the other methods^15^. Furthermore, the FOI is often used urgently as a rescue technique when other intubation methods fail^5,11^. Therefore, reducing intubation time by applying the McIvor blade during FOI is of great clinical significance.

The success rate of FOI is reported to be approximately 88–100%^3^. Similarly, in our study, all cases were successful except for two cases in the J group: one in which fiberoptic bronchoscopy failed to enter the trachea and the other in which tube advancement was not successful. Although the failure rate was not significantly different between the two groups in our results, FOI failure in emergencies can cause significant complications and lead to a grave prognosis in each patient; we have to try to improve the intubation condition by all means possible^16^.

The jaw-thrust maneuver is the simplest and most commonly performed method to clear the airway. In previous studies, this maneuver significantly improved airway clearance at the epiglottis level during FOI^8,10,14^. Furthermore, jaw-thrust does not damage the airway tissue and does not add obstacles to airway management during FOI^8,17^. Therefore, we performed a jaw-thrust maneuver in the control group. In our protocol, the McIvor blade could retract the tongue base forward, lift the epiglottis upward, and secure airway clearance at the epiglottis level^12^. The degree of airway clearance at the epiglottic level was not significantly different between the two groups. However, since the tongue body was retracted together by applying the McIvor blade, airway clearance at the soft palate level was significantly higher in the M group than in the J group.

Lingual traction is also a frequently used airway clearance method for FOI^18^. Pulling and holding the tip of the tongue with gauze or Duval forceps showed a significant airway clearance effect at the soft palate level; however, it did not show proper airway clearance at the epiglottis level^6,18,19^. Therefore, it is recommended to use a combination of jaw-thrust and lingual traction to secure airway clearance at both the soft palate and epiglottis^6^. However, to apply this combination, two or more assistants are required to perform both maneuvers simultaneously, which is frequently impossible in emergency situations. Furthermore, lingual traction can cause tissue injury, and sore throat is reported to be more severe when lingual traction is applied^19^. In contrast, in our protocol, there was no significant difference between the two study groups in the incidence of sore throat.

Intubating the airways and a direct laryngoscope can be used to enhance airway clearance during FOI^7,11,14^. However, these devices are relatively bulky and may impede tube advancement and bronchoscopic manipulation^9^. On the other hand, a thin and flat blade might be more effective in securing sufficient space for manipulation during FOI. Recently published difficult airway management guidelines and case reports have suggested that combined use of video laryngoscope and FOI increased the success rate of intubation in difficult airway^20–22^. However, most of them recommended the use of the fiberoptic scope as a dynamic stylet rather than for a FOI process^22–24^. Combinational use needs at least two physicians to perform the process and is usually utilized as a last resort for extremely difficult cases rather than a routinely airway securing method to facilitate FOI^25^.

Since the McIvor blade could endeavor the airway clearance at both the soft palate and epiglottis levels, the range of insertion angles for tube advancement was further increased. The most common problem encountered when the endotracheal tube advances during FOI is that the tube becomes impinged into the right arytenoid cartilage^26–28^, which is more likely to occur when the tube advances steeply. In our results, fewer tube rotations were required during the tube advancement process in the M group than in the C group.

Recently, Cao et al. invented a new tongue root holder for the anterior displacement of the tongue during FOI and showed that the airway clearance was better when they applied it with a jaw-thrust maneuver than when using jaw-thrust alone^2^. Meanwhile, our protocol utilized the McIvor blade without the jaw-thrust maneuver, which required only one assistant and presented a superior condition for FOI in comparison with the jaw-thrust maneuver. Furthermore, the McIvor blade is a commercialized instrument already available in every hospital that performs otorhinolaryngology surgery.

Our study had several limitations. First, the anesthesiologists and assistants who participated in anesthetic induction were not blinded to the airway clearance maneuvers. However, in every FOI trial, all medical staff did their best to optimize and perform the FOI condition promptly and safely. On the other hand, postoperative sore throat and physical examination data were assessed by a blinded anesthesiologist. Second, we did not include patients with craniofacial anomalies or cervical spinal diseases. Since the possibility of performing FOI is relatively high in these populations, the efficacy of auxiliary devices, such as the McIvor blade might be more critical. Furthermore, the maneuvers we performed in this study might not be applicable in some patient groups. A McIvor blade cannot be placed in patients with limited mouth opening. Applying a McIvor blade during awake fiberoptic intubation might be also challenging. Jaw thrust maneuver could not be effective in patients with limited neck movements^29^. Nevertheless, efforts to improve the success rate and quality of tracheal intubation are of huge clinical importance. In this study, we demonstrated for the first time that the McIvor blade might improve the condition for FOI, compared to the jaw-thrust, the most commonly applied maneuver during FOI. Further studies on the use of McIvor blade in patients with difficult airway or unfavorable conditions should be followed. Third, we should consider the possibility of airway injury caused by tongue retraction. It was reported that subacute tongue edema could occur in the tongue due to the pressure exerted by a McIvor blade^30^. Fortunately, there were no cases of tongue edema and postoperative throat pain was comparable between the two methods in this study, which seemed to be because of the short applying time. Fourth, we failed FOI in two subjects, and their data could not be included in the analysis of intubation time and attempts of tube advancement. However, both patients were in the J group; the exclusion did not significantly depreciate our conclusion. Finally, our study was conducted only in a single medical center, and further studies are needed to increase its generalizability.

In conclusion, this randomized controlled clinical trial demonstrated that applying the McIvor blade showed a superior effect in securing the airway for FOI and shortened the total intubation time in comparison with the jaw-thrust maneuver. Our findings may provide additional clues for airway clearance during FOI which has a significant role in difficult airway management.

## Data Availability

The datasets used and analysed during the current study available from the corresponding author on reasonable request.
